# “Challenging behavior” in dementia care: ethical complications of a well-intentioned concept

**DOI:** 10.3389/fpsyt.2024.1485319

**Published:** 2024-12-12

**Authors:** Jonas Barth

**Affiliations:** ^1^ Faculty for Education and Social Sciences, Institute for Social Sciences, University of Oldenburg, Oldenburg, Germany; ^2^ SOCIUM - Research Center on Inequality and Social Policy, University of Bremen, Bremen, Germany

**Keywords:** challenging behaviour, violence, elderly care, ethnography, ethical issues, dementia, sociology of care

## Abstract

Uncommon behaviours such as aggression, apathy or restlessness are described as challenging behaviours in dementia care. On the one hand, this concept describes a practical problem faced by care staff and, at the same time, defines normatively how care staff should deal with this problem. A frequent benchmark here is the dignity of the person in need of care, which caregivers should also respect in the case of challenging behaviour. However, little is known about the normative standards that are effective in practice in everyday care when dealing with challenging behaviour. Researching these can provide information on which standards are actually applied and encourage reflection on which standards should be applied. In view of the fact that challenging behaviour can also be associated with aggression and/or violence in particular, an ethically significant question arises as to what effects the practical handling of such behaviour has on the extent of the willingness to use violence. The aim of this article is therefore to present empirical findings from an ethnographic study that focuses on the interpretation and practical handling of aggressive behaviour of care recipients by the nursing staff. In essence, it will be shown that a professional approach to challenging behaviour helps to prevent people with dementia in need of care from committing violent acts. If this finding is analysed in terms of its ethical implications, the conclusion suggests itself that the exclusion of the possibility of using violence is to be welcomed, since the exercise of violence makes respect for the dignity of another person, if not impossible, at least more difficult. However, it is questionable whether, under such conditions, the renunciation of violence can still be attributed the freedom required to qualify it as ethically good behaviour.

## 1 Introduction

When caring for people with dementia, those in need of care may display behaviours that carers find challenging. Such behaviours can include apathy, restlessness or aggression. It is important for nursing research to explain the emergence of such behaviours and to investigate which nursing interventions have either a changing effect on such behaviours or on their experience (1–3). An important aim of this nursing research is to increase the level of professionalization of nurses by recommending certain forms of interventions (4–7). The recommendations cannot be based solely on the effectiveness and nursing feasibility of such interventions, but must also be able to specify the purposes that these interventions are intended to serve. These purposes are based on evaluative standards that are often taken from nursing ethics.

A central ethical benchmark that is regularly used as an evaluative standard is the emphasis on human dignity (8–10). This usually refers to two things: firstly, care should be based on the principle of respect for the person. Secondly, carers should strengthen the autonomy of those in need of care. It is clear that, based on the generally defined duty to respect the person of people with dementia, specifics are required as to how such respect can be conceived and implemented. A particularly widespread approach to this is the concept of person-centred care (11, 12). While this concept is intended to be the standard for all care situations, specific care situations, such as those involving challenging behaviour, require specific concretisations that explain precisely what constitutes person-centred care that respects dignity (13).

However, as important as it is to standardise nursing practice on the basis of evaluative benchmarks and feasibility studies, such specifications say little about the actual interaction in such situations. Examining those is not only relevant for determining the extent to which the assumed standards are correctly implemented in nursing care, but can also provide information on the extent to which the assumed standards, if they are implemented, fulfil their purpose at all. Secondly, such studies can determine the extent to which - whether due to practical necessity or other reasons - other standards may apply, i.e. which evaluative standards are actually used by nursing staff to interpret such situations and derive practical consequences (14). Findings from such studies can in turn contribute to reviewing and, if necessary, correcting assumed ethical standards of nursing behaviour. The actual practice of dealing with challenging behaviour can be examined in different ways. Considering that challenging behaviour can also be associated with aggression and/or violence in particular, an ethically significant question arises as to what effects the practical handling of such behaviour has on the extent to which it leads to violence.

Studies that focus on the *interpretation* of aggressive behaviour of care recipients are not yet common (15–17) or are still in the planning stage (18). The interpretations have so far been recorded on the basis of interviews. Interaction studies based on participant observation have not yet been carried out with this focus. While it has been researched, for example, that nursing staff attribute aggressive behaviour of people in need of care to different causes, it remains unclear what consequences the nursing staff draw from these interpretations and what consequences this has for the normative order in care as a whole. However, for the ethical evaluation of the nursing approach to challenging behaviour, it is particularly important to include the consequences of dealing with the aggressive behaviour of people in need of care in the research.

To this end, the findings of an ethnographic study will be presented in which situations of challenging behaviour were observed and the resulting observation protocols and interview transcripts were evaluated in the style of Grounded Theory Methodology (19) (section 2). In essence, it will be shown that a professional approach to challenging behaviour helps to prevent people with dementia in need of care from committing violent acts (section 3). If this finding is analysed in terms of its ethical implications, the conclusion suggests itself that the exclusion of the possibility of using violence is to be welcomed, since the exercise of violence makes respect for the dignity of another person, if not impossible, at least more difficult. However, it is questionable whether, under such conditions, the renunciation of violence can still be attributed the freedom required to qualify it as ethically good behaviour (section 4).

## 2 Materials, methods and theory: ethnography, grounded theory and phenomenology

### 2.1 Social theory: a reflexive understanding of violence

If one examines interaction situations with regard to violence, one can ask, for example, how violence affects the course of interaction or, conversely, how a course of interaction can contribute to the emergence of violence. In both cases, however, it must be assumed on the part of the observer what is meant by violence and what is not. In addition, violence is usually framed normatively as undesirable on the basis of such a preconception. Two different strategies for defining violence are common in the social sciences, which cannot be linked to each other, but which are similar in the way they are used as described above. A narrow understanding of violence (20) emphasises the restriction of violence to the injury of another person’s body. A broad understanding of violence, such as ‘structural’ (21) or ‘symbolic’ (22) violence, on the other hand, relies on forms of suffering that are analogous to violence. While studies on violence in the care sector have so far focused on these concepts of violence (23, 24), a different approach is necessary when analysing interaction situations in terms of how they determine what constitutes violence (or the absence of it) and how legitimate and illegitimate violence are distinguished. I propose a definition of violence as a ‘reflexive’ understanding of violence, which begins with the interpretative practices of those being analysed (25–30).

It is rooted in phenomenological thinking and based on the proposal to understand violence in connection with harming and suffering as an institutionalised context of order (31–35). The understanding of violence is based on the principle of mediated immediacy (36). Accordingly, violence is characterised by the direct experience of a lived body (German: *Leib*) in harming and suffering (25). The experience cannot yet be considered violence because it must first become recognisable as such. It does so insofar as it is always mediated symbolically and communicatively. This means that in addition to the dimension of the lived body, the discursive dimension of violence must also be included in the analysis. Drawing on the distinction between normative and cognitive expectations (37) violence is used to make normative claims insofar as the use of violence symbolically expresses that certain expectations are upheld even in the event of disappointment. Whether it is violence in a particular case, what distinguishes it in terms of content, whether it is legitimate or illegitimate, is not only dependent on the interpreter, but must also exist as a representation to third parties, insofar as only this reference ensures that the interpretation cannot be arbitrarily revised, but can be socially generalised, i.e. exist as an institution.

Thus, violence is present when actors are involved in an engaging antagonistic lived bodily interaction in the context of harming and suffering, this relationship is communicatively and symbolically interpreted as (il)legitimate violence, insofar as the validity of normative expectations is represented in the antagonistic interaction and this interpretation is claimed as valid with reference to the expected expectations of third parties (26).

Even if this understanding of violence also places the lived bodily dimensions of harming and suffering violence at the centre, this does not yet imply who can be the author or addressee of violence. In the sense of the ‘social undecidedness relation’ (38) a decision on this question is left open and made researchable with reference to violence, because violence - understood as a phenomenon in terms of mediated immediacy - is precisely a representation of who is its addressee and originator of violence in a specific situation. This is precisely why violence is coextensive with the expansion of the normative: Only those who are considered moral actors can exercise or suffer violence, and vice versa: only those who may exercise or suffer violence can be moral actors.

### 2.2 Data & methods: ethnographic research & reconstructive analysis

The data used for this article is based on ethnographic field research (39), which I conducted over a period of approximately 6 months in 2016. During this time, I took part in professional dementia care as a participant observer in two different residential care facilities in Germany specialised in professional dementia care. The main reason I needed a second care facility was that I could better anonymize actors and their actions. An additional benefit resulted from using the second device for the investigation of contrasts (for the use of contrasts see below). I obtained informed consent for the field research from all participants. Participation enables researchers to ‘play along’ in the field and it promotes trust, which can be exchanged for further observation opportunities. The observation data in the form of handwritten notes made on site and repeatedly discussed with field participants were always digitised promptly and converted into ‘observation protocols’ with the lowest possible degree of interpretativity and the highest possible degree of descriptiveness. These observation protocols are the actual data for the analysis and are around 400 pages long. In the context of ethnographic research, further data sources can be tapped, and I have followed an opportunistic understanding of data (39): Data can be anything that appears useful in revealing the rules of a field. In addition to the observational data, I used data obtained from open guideline interviews, the aim of which was to make care-related experiences the starting point for episodic narratives. These were conducted in 2016/17 with 8 carers and 2 people in need of care and lasted between 45 and 120 minutes each (for this article, I have only used the data from the interviews with the carers). They were recruited on the basis of participant observation. This means that - as in the observation protocols - I spoke to the nursing staff on a first-name basis and to the people in need of care on a second-name basis. Accordingly, the nursing staff were pseudonymised with first names and the patients with surnames. The data was not made available to a repository and are held by the author. While the data were originally collected in German I translated them into English.

An important goal of ethnographic research is to reveal the rules of the field - its methodicity (40). The data obtained must therefore be analysed in such a way that the analysis leads to theoretically abstract statements about the field being researched. Establishing such a theory rooted in the object that is analysed is the declared aim of grounded theory methodology (19), which is why I have modelled myself on it. Its core features include:

1. It provides for a successively abstracting coding process that takes place via the constant comparison of formed concepts. However, the sequence of open, axial and selective coding is not a schematic process, because:
2. With the concept of theoretical sampling, data collection and data analysis basically follow an iterative-cyclical process (41). Data collection and data analysis are based on the principle of minimum and maximum contrast. This distinction replaces the distinction between verification and falsification in that the replicability and limits of concepts and categories are checked along minimal and maximal contrasts between different cases and so the theoretical integration of the data may advance. The sampling strategy therefore always includes the request to search for new minimum and maximum contrasts until no more are found (theoretical saturation). Following this line of reasoning, the amount of data is less relevant than its theoretical instructiveness in the research process. According to the task in finding and creating contrasts, the observations are to be validated by the participants even though the validation process did not follow a participatory design (42). My observations were validated (or even falsified) in three not clearly differentiating ways: a) Later observations of similar situations might have shown similar or contrasting outcomes b) As is usual for participant observation, I was in constant communication with the participants, trying to validate my observations and thoughts. But these communications are nothing else then new observations c) I conducted interviews with participants and I observed them in ‘natural’ situations how they communicate with me or each other to let them show to me their own relevancies of how to interpret certain situations.
3. Like any other primarily inductive method, grounded theory methodology also provides for theory-guided coding, as long as the terms used fulfil the purpose of opening up material interpretatively and not assigning it to theoretical premises based on subsumption logic. To this end, they must be as empirically insubstantial as possible (43). The place for such theoretical premises in the grounded theory methodology is the coding paradigm, which can be changed depending on the requirements of the research. For this purpose, I used a coding paradigm specially developed for the sociological research of violence based on a reflexive understanding of violence (26), which combines the theoretical premises mentioned above with the demands of qualitative-reconstructive research.

While the research logic is inductive, the presentation of the results follows a more deductive logic: the aim is to present essential elements of a theory about the field, i.e. the key category and some subcategories, by substantiating central assertions with the material.

## 3 Results: Why persons with dementia may not act violently

Three findings are presented below. Firstly, the key category is presented. This is a pattern of interpretation^1^ whose effect is that people with dementia hardly ever commit violence, but can easily suffer violence (3.1.). This pattern of interpretation contrasts with another pattern of interpretation according to which carers *ad hoc* assume that people with dementia are capable of and intend violence. On closer inspection, this is an action problem that carers have to solve so that they can continue their work (3.2). The problem of action consists of how carers can successfully prevent themselves from applying the latter pattern of interpretation. A number of strategies have been established in nursing care for this purpose, which therefore function as subcategories of the key category. One will be presented in this article: The administration of psychotropic drugs is intended to ensure that care recipients do not exhibit behaviour that they could interpret in terms of the undesired interpretation pattern (3.3).^1^

### 3.1 Why people with dementia cannot perpetrate violence, but can suffer it

The pattern of interpretation institutionalised in the inpatient care of people with dementia is characterised by a three-step logic: The starting point for activating this pattern of interpretation is the production of assaults by a person in need of care that are experienced by a caregiver, i.e. so-called challenging behaviour. This finding was obtained in the interpretation of reactions to a care situation presented to nursing staff that was taken from another care organisation:

Mrs W. was mobilised to the edge of the bed in the morning as usual. Mrs W. hit, kicked and swore at the carer. The geriatric nurse (in the following GN) spoke to her slowly, gently, in short sentences and calmly. Despite her illness, the nurse tried to explain her actions as simply as possible. GN held her gently by the arms to prevent her from falling. Mrs W. was very active in this situation, stood up more often and was unsteady when walking. GN also tried to avoid further blows by holding her arms more tightly. [ … ] The GN ‘s thoughts in this situation were to deal with the basic care as quickly and comfortably as possible. GN had sympathy for Mrs W. and was able to empathise with her situation. However, there was also the thought that basic care had to be provided (e.g. due to incontinence), even if the resident showed this defensive behaviour. The actions in the situation were that the nurse tried to work even faster, as well as to continue to avoid kicks and blows, to have a calming effect on Mrs W. and to talk to her about other topics such as the weather. After care, Ms W. was mobilised into a walker, in which she usually calms down and ‘only’ grumbles to herself. GN then takes Mrs W. to breakfast and lets her rest there.

The quality manager interviewed commented on this as follows:

I would consider it a successful situation. #mhm# So the carer is right. Mrs W. shows that she did not want to be cared of. But that is the pathological change. To what extent Mrs W. would also have decided, if she had been clearly conscious and heavily soiled, not to let herself be helped to clean herself again #mhm# can be answered clearly in most cases: None of us walk around like that voluntarily. (Interview GN B).

This answer contains the first two phases of a three-step logic of interpretation:

1. The quality manager interprets Mrs W’s behaviour ‘that she did not want to be cared of’.
  A carer interprets this behaviour *ad hoc* as a communicatively meaningful action, the intention of which is not to want to be cared for. If this communicative expression were taken seriously, it could have been a violent act.
2. However, this attribution of intention is immediately relativised: ‘But that is the pathological change.’
  The GN recognises that it is a person with dementia and attributes the assault to the dementia. This relativises the intention. The assault therefore appears to be involuntary. It can therefore no longer be violence that communicatively expresses normative expectations. The first intention is then replaced by a generalised intention of wanting to be cared for in any case, which would presumably be the case from the perspective of the GN if the person did not have dementia. However, as the person has dementia, she cannot introduce their intention into the flow of communication. The fact that this is non-communication and therefore automatically also non-violence is not interpreted arbitrarily, but requires reference to legitimising third parties.
3. At another point in the interview, the quality manager completes the three-step process:

So action must be taken. The person must be helped, she must be cleaned up to prevent other damage, skin damage etc. #mhm#. These are things that are absolutely necessary. (Interview GN B).

The duty to provide care is derived from the relativisation of the initially understood intentions of the person in need of care. Acting contrary to this obligation as well as using violence that is not necessary for this purpose is considered illegitimate violence. The GN must present themselves to various third parties in such a way that there is no reason for this interpretation. The difference between interpretation step 1 and interpretation step 2 corresponds to a judgement about the actor status as well as about the commonality of interpretation steps 1 and 3: Someone whose external behaviour is denied intentionality due to a permanent illness cannot (any longer) be expected to be able to establish a consistent relationship between their own will and expression. Anyone who is unable to do this can no longer communicate and therefore cannot use violence. The fact that GN ‘s are under pressure for their behaviour to be interpreted as illegitimate violence is not least due to the fact that a) they expect that they are expected to establish a consistent relationship between expression and intention, and b) that in interpretation step 3 they put themselves in a situation in which, conversely, residents find themselves in interpretation step 1: as potential perpetrators of violence.

The intention of not wanting to be cared for is inferred from the initially observed physical behaviour. This intention is relativised and the behaviour is interpreted as the involuntary expression of a state of illness, whereby the behaviour must also be overcome by force in case of doubt, insofar as this is associated with the violation of the resident’s physical well-being. On the basis of this interpretation of physical behaviour, it is impossible for Ms W. to use violence. Nevertheless, the presentation of this sequence of interpretations fails to recognise the difficulties for care staff in applying this pattern of interpretation. The application of the interpretation pattern, which relativises the intention of violence on the part of the person in need of care, corresponds to the fact that the carers must take care not to allow themselves to be injured.

### 3.2 Opportunities and limits for carers to make themselves invulnerable

It is not at all the rule that all people with dementia regularly display behaviour that can be interpreted as violence. However, *if* it does occur, carers have to update their professional interpretation routine against other possible interpretations. To this end, they try to dethematise or play down violations of norms. In direct care, however, there are situations in which this strategy does not work. Based on their own direct experience of the situation, which they sometimes experience as a potential illegitimate experience of injury, they may use violence to represent the inappropriateness of the resident’s behaviour.

Caring for Mr Kaiser is a particular test. Carers usually provide care in pairs. They *expect* that they will have to be prepared to provide a difficult care for Mr Kaiser. They steel themselves internally, try to develop a specific attitude and are nevertheless caught up in a dynamic in which they develop and apply a pattern of interpretation that ascribes specific intentions of violence to Mr Kaiser. This puts them in the difficult position of having to put two competing patterns of interpretation into a practical relationship with each other, because completely different reactions are appropriate to violence than to forms of behaviour that only outwardly resemble violence but are in fact involuntary symptoms of illness.

The following is a description of a care situation with Mr Kaiser:

The two of us go into his room. ‘Oooh,’ says Ruth. ‘I can’t stand it in this room.’ It really stinks terribly. She tells me she doesn’t know if I have to go in with her. I could also stand in the doorway. When I ask her, she confirms that the smell is just urine. ‘Good morning Hans,’ calls Ruth. She goes to him in the bathroom and wants to pour water into a plastic tub. It rattles loudly. I ask if everything is OK and open the door to the bathroom. She swears and tells me that the soap holder has fallen off the wall. Kristina comes in and asks who will do the body wash. Ruth suggests that they both wash at the same time. One on top, one on the bottom of the body. Kristina agrees. She throws back the blanket. ‘All full!’ she shouts. Ruth pulls off the duvet and throws both into a plastic tub. Turning to me, she says that’s not really the way to do it. ‘Eeeh’ shouts Kristina. Both GNs are visibly disgusted by what they find. ‘The diaper is dry,’ they exclaim. The urine is up to the shoulder. They are puzzled as to how Mr Kaiser has managed to keep the adult diaper dry while soiling a large area with urine. They refrain from answering.

Mr Kaiser pinches and punches Ruth. Ruth shouts: ‘Hitting is bad.’ Ruth shouts that they just want to wash him. ‘No!’ he shouts. But this refusal is not taken up any further. Meanwhile, Kristina runs to the door and closes it. She doesn’t want the quality manager to come in and see her. Then she would quickly lose her job. She says this in a mixture of seriousness and an ironic undertone. She goes back to Mr Kaiser and dresses him while Ruth holds his arms. Ruth tells him not to be so ‘angry’. If someone has a reason for violence, she can understand that. But with him it is ‘pure malice.’

Mr Kaiser is now sitting in the care chair. Kristina has shaved him. She then approaches him with a plastic cup and toothbrush. He knocks the cup out of her hand. The water in the cup splashes in all directions. ‘Oh, you arseh…’ shouts Kristina, but breaks off in mid-word. Together with Ruth, she realises that she actually would have wanted to shout: ‘Oh, sheesh!’ I have to laugh at that. I have the feeling that they are both overwhelmed by the situation. Ruth takes Mr Kaiser to the dining room and Kristina tidies up the room. I go to the dining room too.

Kristina walks past me and tells me she hopes it wasn’t too bad. I wonder for whom. I appease her and tell her that I’ve been to see Mr Kaiser before. Shortly afterwards, Ruth comes by and laughs at me, saying that Kristina is now walking all bent over because she is so unsteady.

A few minutes later, I overhear a snippet of a conversation between Ruth and Dirk about Mr Kaiser. Ruth says: ‘He’s mean. He’s really mean!’

It is not the case that all of Mr Kaiser’s care is provided in this way or so drastically. In any case, it is the case that the carers are prepared for it to take place in this way.

Immediately after the care begins, the carers and Mr Kaiser enter into an antagonistic relationship, but this does not lead to the carers stopping the care. At least Kristina expects that the carers’ behaviour could appear to be a case of illegitimate violence from the perspective of the quality management. In fact, it is not common for carers to close the door and thus exclude the presence of third parties. This indicates that the standards of legitimacy that the quality manager and Kristina apply are not the same in Kristina’s eyes.

The nursing staff are not sure as to whether they should interpret Mr Kaiser’s behaviour as illegitimate violence. For example, the nurse Kristina uses the interpretation pattern explained in 3.1 in relation to Mr Kaiser:

‘Yes. Erm (sighs). (4) That’s on the agenda. You come in, say good morning and sometimes instead of good morning you get slapped. Or you’re brutally ignored by a resident. And the more active you become, the more you talk, the more the resident gets angry and can also become physically active - in terms of hitting and kicking. [ … ].

I can only say that perhaps you have noticed that Mr Kaiser also cries a lot and often? #mhm# It doesn’t matter whether he’s very sweet or aggressive, it has to do with his stroke. He probably can’t control it any more. #mhm# I suppose this aggressive behaviour too, the clinging to us and hitting. Maybe that’s why he can’t control it either. That’s what the doctor said about the crying, because we also presented the whole thing to the neurologist. Because we didn’t know whether we were causing him pain or what. But I can imagine that he can no longer really control his behaviour, his aggressive behaviour.’ (Interview GN A).

The case in the quoted observation is different: according to Ruth, Mr Kaiser is ‘evil’ and ‘mean’, which suggests that his actions are not involuntary and that he has intentions to hurt, for which Ruth cannot recognise any legitimising reasons. Kristina’s spontaneous exclamation that Mr Kaiser is an ‘arseh…’ also speaks in favour of an *ad hoc* attribution of intentionality: An arsehole is always someone who decides in favour of a certain alternative course of action, knowing full well that other *possible* alternative courses of action do not cause this harm to other people. An arsehole therefore at least accepts the harm to other people, even if it is not clear whether they are doing this in order to gain a material advantage, for example, or whether they are doing it out of pleasure in the harm itself. The decisive factor is that arseholes would always have had alternative courses of action.

In the following scene, the interpretation that Mr Kaiser’s behaviour is violent is supported by excluding alternative interpretations of violence: ‘Ruth shouts that they just want to wash him.’ This is a sentence that initially supports the above-mentioned interpretation that Mr Kaiser has no legitimate reasons for his behaviour. This becomes clear with the adverb ‘only’: Ruth anticipates the possibility of evaluative comments on her behaviour. The content of her behaviour consists of the intention to wash Mr Kaiser. With regard to this content, from her perspective - this is indicated by the ‘only’ - a negative evaluation is not to be expected. She thus doubts the possibility that the pinching and hitting constitutes such a statement and, accordingly, her behaviour does not appear to her as behaviour that is normatively criticised. Against the background of the assumption that there must be sound reasons for the use of violence, the ‘only’ excludes the possibility of such reasons. This also explains why Ruth does not respond to Mr Kaiser’s exclamation ‘No!’: It is already established that Mr Kaiser cannot provide any acceptable reasons for the negative evaluation of Ruth’s behaviour.

From the nursing staff’s perspective, it is impossible for their care activities to constitute violations of norms for Mr Kaiser. Mr Kaiser’s normative claim cannot be based on this. The care situation described above clearly shows that there is no need for this: The shout ‘No!’ towards Ruth and his assault allow in principle the interpretation that the nursing staff have committed norm violations towards Mr Kaiser - but they do not claim it. By attributing malice to Mr Kaiser, the normative claim made by Mr Kaiser is reduced to his self-assertive right to use violence whenever it is at his will.

Against the background of such an interpretation pattern, nursing staff are faced with a difficult situation: if they maintain this interpretation pattern, they evaluate the behaviour of the person in need of care, i.e. they have to decide, for example, whether Mr Kaiser is *allowed* to act in this way. As they are the ones who are directly affected by his actions, they are also the ones who have to demonstrate a negative evaluation of his behaviour to him in a communicative manner. In principle, there are different ways of presenting this behaviour. Kristina’s exclamation: ‘Oh, you arsehole…’ is the beginning of the use of such a possibility - it is not only an interpretation of Mr Kaiser’s behaviour as an act of violence, but also an evaluation of it: this shows that Mr Kaiser’s behaviour violates norms and that Kristina is also affected by the disappointment of expectations.

However, if carers act as evaluators on the basis of such an interpretation of violence, they also present this evaluation to third parties. They must therefore also anticipate with regard to third parties whether the form of their evaluation can be expected to be judged as appropriate. One type of third parties is the second carer present. Kristina closes the door in order to exclude other third parties and thus competition from third party’s different perspectives. Incidentally, this is a strategy that is not without risk because closing the door may still be visible to third parties: For example, if someone observes the closing process because noises can be heard from the room or because the presence light on the outside above the door is switched on. The fact that in this case the evaluation ‘arsehole…’ is nevertheless made in front of possibly competing third parties probably occurred to Kristina during the utterance, which is why she stopped it. It is unclear here whether the result of the consultation with Ruth, that she wanted to shout ‘sheesh’, applies to me as the observer present or, for example, serves to make amends for the shock about herself. Both together seem plausible, above all because Ruth’s statement that Kristina is now walking ‘all bent over’ suggests that Kristina is obviously evaluating her own behaviour negatively, assuming how I would judge it.

This dynamic of spontaneously interpreting a behaviour as illegitimate violence and that the response is likely to be behaviour that falls under the same interpretation is confirmed and supplemented by another carer:

So boundaries that should not be crossed are, um (3), um, unnecessary physical violence. # mhm# Um, the fact that you might have to hold tight a resident’s hand or foot to avoid being kicked is still understandable for me. But if you suddenly feel the need to slap that person in the face or something like that, that would definitely be crossing the line. Erm. Is not okay at all. But unfortunately, from my point of view, you always work very, very close to it. Residents can be very, very provoking and you really have to be careful not to cross that line. Verbal abuse is another nasty thing but that needs to be interpreted a bit more generously. Um insults wouldn’t necessarily be favourable or aren’t favourable. But it has been shown that in some situations that have occurred, clear, loud words have led to success. #mhm# A kind of commanding tone, yes, that such things have actually led to success. [ … ] So I think the verbal aspect has definitely reached its limit when you get into insulting behaviour, because I can’t imagine that insults will probably lead to success. #mhm# Physically, if it turns into unnecessary violence and, uh, verbally, if it goes somewhere insulting, which makes no sense. #mhm# # (Interview with GN D).

‘Residents can be very, very provoking and you really have to be careful not to cross that line.’ The spontaneous tendency to interpret challenging resident behaviour as intentional and illegitimate is described here. This can lead to ‘suddenly feel the need to slap this person in the face.’ This need to punish presupposes the previous interpretation and activates the communicative representation of the validity of normative expectations. It is interesting that the interviewee now distinguishes unnecessary violence and insults from other forms of assault (holding hands and feet, tone of command). The demarcation criterion that she motivates is performance-related: Does the behaviour displayed lead to success? For example, the commanding tone can motivate a resident to co-operate. The holding of limbs serves to maintain care without the carer having to accept injuries. In order to be able to differentiate between unnecessary violence and legitimate coercion in this way, however, it is necessary to ensure that the resident’s behaviour is no longer interpreted as illegitimate violence that needs to be evaluated, but rather, for example, as a disturbance that needs to be overcome or circumvented.

Against this background, steps must therefore be taken to switch to a pattern of interpretation that makes it unnecessary to act as an evaluator of violence at all. Strategies for this can start in two places. Firstly, carers can start with the way in which they directly experience the behaviour of those in need of care. In this sense, carers cultivate a habitus of invulnerability. Pain-avoiding postures and turning away from those parts of the body in relation to which the normative expectation of pain is particularly obvious help carers to avoid acting as evaluators of violence:

‘[W]ith time, you even develop postures. Somehow it develops that you can’t be hurt quite so much. You watch how you present yourself. It’s very important to protect the facial area #mhm#, whether you wear glasses or not. I find nothing worse than being hit in the face. But also: you really develop grips. Maybe that sounds really brutal now, but you develop grips, you develop a stance so that you don’t hold on to the resident too tightly, but are a bit distanced from your body and can still work.’ (Interview with GN A).

Secondly, however, the behaviour of people in need of care can also be used to stabilise the fact that they do not (or cannot) have any intention of harming others. For example, nursing staff attribute Mr Kaiser’s behaviour to illness and thus eradicate the action character of his behaviour. However, this strategy only takes place ex post and thus continues to carry the uncertainty of other behavioural interpretations with it. One strategy to ensure that behaviour that could activate the first interpretation pattern does not occur in the first place is to administer psychotropic drugs. This is discussed in the following section.

### 3.3 Psychotropic drugs: ensuring non-violence in care

The use of psychotropic drugs in the care of people with dementia is often criticised. Depending on the form of dementia and the drug, it may be medically contraindicated (44, 45) or it may increase other risks, such as the risk of falls or other (46, 47). Accordingly, the guideline in Germany is to minimise their use as much as possible (48). This contrasts with findings of health care sciences that dementia patients are, at least in Germany, under-supplied with anti-dementia drugs and over-supplied with antipsychotics both in home and inpatient care (49). This is attributed, for example, to the use of on-demand medication (50) or to the decline in (other) measures involving deprivation of liberty, such as the use of bed rails (51).

It is therefore not surprising that the administration of psychotropic drugs is sometimes referred to as ‘chemical violence’ (52) or ‘chemical restraint’ (53). One *sociological* explanation for their use is that the administration of psychotropic drugs critically controls the potential for violence on the part of nursing staff by reducing the likelihood that the behaviour of a person in need of care will be interpreted as (illegitimate) violence in the first place. I would like to illustrate this with a case in which a person in need of care with dementia receives successively increased doses of the psychotropic drug Melperon, initially via the on-demand medication, but then also via the neurological prescription.

The case is about Mrs Pete, about whom care staff initially noted: ‘Mrs Pete has settled in, approaches fellow residents and GN.’ Eventually, however, they changed their minds:

Telephone call to Löwith’s practice, asked to be called back. Very noticeable behaviour since the weekend. She is tearful, caught up in her negative marital experiences, talks about them. She can’t be distracted by anything, then gets angry, insults coresidents and misjudges situations. She irritates other residents with insults and intrusive behaviour, thereby endangering herself. (Observation protocols).

After about two months, which Mrs Pete had already spent at the residence, she apparently developed a behavioural disorder, which prompted the nursing staff to consult the neurologist and successfully request a change to the prescription for the on-demand medication. As a result, the long-term medication was extended to four doses of Melperon per day and the on-demand medication developed into continuous medication, which was medically sanctioned and finally supplemented with the neuroleptic Quetiapine. What happened?

The change in medication was a reaction to several events that had taken place since the weekend, according to the entry in the documentation. At least this is suggested by entries in the so-called handover book:

Entries about Mrs Pete. She had refused food a few times. It now also says when she accepted food. She has often insulted people [ … ] She has also threatened to hit them and last night she even hit Marion with her fist. Her legal trustee and partner visited her yesterday. Afterwards, she was ‘even more angry’. (Observation logs).

The eating behaviour, insults, threats of beatings and the one-off beating of a carer provide initial indications of what might have made the medication change necessary from the carers’ point of view. The following is a very abbreviated description of a situation in which Mrs Pete was involved and which subsequently triggered a series of reflections among the carers:

I go into the nursing home and meet Mrs Pete and Mr König in the seating area between the staff room and the large dining room. I shake hands with both of them. As I shake Mrs Pete’s hand, I notice that she is very upset. She was already in a bad mood last week. But today she seems to have hit rock bottom. She says she doesn’t want to eat. She keeps getting up, standing in the passageway to the dining room or changing her seat in the aforementioned seating area. This consists of a corner bench and two leather-covered armchairs as well as a small round table. Passing GNs are drastically insulted: ‘You fat bastard’, ‘arsehole’ etc. She says about Anna, also a carer, that people say she’s a beauty. But Mrs Pete is sure: ‘She’s wrong.’ Shortly afterwards: ‘I feel puke-sick!’ The GNs ignore her or make eye contact with me instead. They raise their eyebrows or roll their eyes. There is no evidence that Mrs Pete changes her behaviour in response to the reaction she elicits from others [ … ].

Mrs Pete is a topic of conversation in the break room. Everyone agrees that Mrs Pete’s mood has worsened since the beginning of last week. Heike opts to give her a tranquilliser because she can’t find her way out of this aggression on her own. Anna agrees. I describe my impression that she is mixing up current events with things that happened a long time ago. Heike says she once learnt that dementia is like a shelf of books. Each book represents a year of life. With dementia, all the books fall over starting from the back. [ … ] (observation protocols).

Mrs Pete’s insults are interpreted as pathological. Maria answers my question about whether the strain on carers is always the same for mobile residents:

No, for some people it’s higher. Erm, Mrs Pete through her insults, but she probably can’t help it, because she might say this insult against another person who is still in her head, a kind of Tourette. (Interview with GN D).

Nevertheless, these are also seen as a normative problem:

Mona comes by and gets loud. She shouts that Mrs Pete can vent her bad mood in the entrance area. But that’s not possible at this place because: ‘We’re a community here!’ (Observation logs).

However, it is the task of this community to ensure that people are not excluded from it. Mona discusses this in an interview:

Interviewer: I can remember, for example, that there were many discussions about this with Mrs Pete. #Yes# Um, whether to increase the medication, whether it was enough, whether she was well adjusted or not, whether to give her more time or not. #Mhm# Um, how did that go?

Mona: Then you’re exactly on point. Um, this woman certainly had needs and fears. #Mhm# Inside herself. And couldn’t handle it any other way than the way she always reacted: With swearing, ranting, insults. Until some other resident was possibly no longer able to control it and would have endangered her. #Yes# And so we then discussed in the team, part of her reactions is certainly character-related, biography-related, um and perhaps with the help of the neurologist and medication the whole thing can be dampened down so that she no longer suffers from it and doesn’t endanger herself by perhaps causing others to beat her. #Mhm# And the colleagues are always different in their um, in their ability to put up with it. Some clearly see what I said, that it’s character-related. You can’t change some things. Erm. And the others think that if I put something on top, then we’ll have peace but that’s not our approach. What, I can understand that too. It’s not a job that can be taken lightly. #Mhm# (2) But you are exactly on the point of what I mean. The person who is then given medication should be fine. #Mhm# Not to flatten him and make him quiet, to make him compliant, but to make him well. #Mhm# And that has to be communicated to everyone involved. (Interview with GN C).

Mona confirms Heike’s interpretation that Mrs Pete suffers from her own aggression and confirms the task of the nursing staff to eliminate this suffering with the help of medication by ‘damping’ it. Even if the existence of a pathological condition and the pressure of suffering are not sufficient to justify the change in medication, they are included in the justification as a purpose. Mona now adds further purposes to this: Mrs Pete’s social identity, the benefit calculation of carers and the preservation of the normative order of her community. She fears that Ms Pete’s behaviour is harming her in a completely different way than just the fact that the aggression itself is already causing psychological strain: Mona anticipates that Ms Pete’s behaviour is a provocation for others and that these others could resort to violence in response to this provocation. The help that the GNs want to give Mrs Pete is therefore twofold: firstly, they free Mrs Pete from suffering from herself and, secondly, they prevent her behaviour from giving others an opportunity to use violence to demonstrate the validity of their disappointed expectations, so that Mrs Pete ‘doesn’t endanger herself by perhaps causing others to beat her’. Interestingly, Mona mentions this latter motive for help twice, but only refers to residents in the first case, leaving it open the second time. At least implicitly, Mona reveals an understanding of violence here that seems to assume that GNs could also be put in the situation of exercising violence. In this sense, the medication not only protects Mrs Pete from other residents, but possibly also from GNs. The medication can therefore not be seen separately from the local social order, its applicable or assumed norms.

## 4 Discussion: ethical issues concerning the impossibility of acting violently

The findings presented in this paper are merely indicative insofar as they cannot claim that the patterns found in the data are to be found in every dementia care facility. Nonetheless, the findings presented underline the fact that nursing staff in their professional role tend to pathologise aggressive behaviour of people with dementia in care facilities (54). However, the entire behaviour is not pathologised, but it is more precisely a question of casting doubt on whether the aggressive behaviour has come about of its own free will. With reference to the pathological condition of the people with dementia in question, it is denied that they harbour intentions to harm. However, it is not denied that they have any intentions at all - which would be the case with total pathologisation. This is related to the fact that a generalised intention wanting to be cared for is assumed here. This shall help the nursing staff to interpret the behaviour as an indication of unmet needs and to contribute to their satisfaction in accordance with their nursing skills.

Along the lines of everyday common sense, however, it seems *counterintuitive* to ask carers to adopt an interpretation according to which attacks on their own bodies should not be understood as illegitimate violence. In fact, it has been shown that carers do not readily attribute aggressive behaviour to dementia, but in some cases have great difficulty in distinguishing this pattern of interpretation from an interpretation according to which aggressive behaviour is due to intentions to injure. Insinuating those intentions is problematic from a professional theory point of view because such an interpretation changes the further care interaction in such a way that carers feel disappointed in their expectations and are therefore challenged to explain to the person with dementia which expectations should apply. They can do this, for example, by giving moralising speeches but even by using violence.

Precisely because it is sometimes a great challenge in everyday care to use the institutionally favoured pattern of interpretation, it makes sense to solve this problem in such a way that it does not yet arise. This is the case when people in need of care are prevented from displaying aggressive behaviour in the first place. Seen in this light, it is understandable why the administration of psychotropic drugs helps to prevent this behavioural problem from arising.

If one attempts to guide the professional handling of challenging behaviour ethically in such a way that the dignity of the person behaving in this way is preserved or even promoted, it is not surprising if the latter interpretation pattern is certified as not serving to respect the dignity of people with dementia. However, the institutionally favoured pattern of interpretation also raises at least three ethical remarks.

### 4.1 The dignity status of care staff

By declaring respect for the dignity of those in need of care, respect for the dignity of carers is pushed into the background. As respect for the dignity of those in need of care is a norm that care organisations use to control the behaviour of caregivers, this results in the fundamental problem that claims to autonomy are undermined if they are understood as prohibition of instrumentalization in the Kantian sense (55) but demanded heteronomously: They then become the prevailing morality. Whilst this problem cannot be solved completely, it is possible to deal with related practical problems: If carers experience the behaviour of people in need of care as violence, this can be accompanied by the fact that they feel their dignity has been violated. Insofar as this is not the institutionally preferred pattern of interpretation, this can lead to carers seeing their experience of such dignity violations devalued by care organisations.

### 4.2 The dignity status of those in need of care

Respect for a person’s dignity involves recognising that person’s freedom as a condition of the possibility of their moral actions. Organised doubt about intentions to harm can therefore possibly be reconstructed as a violation of the dignity of people in need of care insofar as their aggressiveness is not attributed to freedom and it is therefore questionable to what extent non-aggressive actions can be understood as actions that make use of autonomy. Should respect for the dignity of people in need of care therefore not also include the promotion of their autonomy insofar as it enables them to decide against aggressive behaviour of their own free will? This thought would be, on the one hand, in line with the concept of person-centred care (12) as it focuses on the recognition of the autonomy of the persons with dementia. But, on the other hand, this concept would have the tendency to judge this view as part of the so-called malignant social psychology insofar as it may be part of a ‘dark’ view on human personality, assuming human people willingly act violently. But – as Kitwood is arguing on the basis of recognition theory – the ethically more challenging question would be, if the concept of person-centred care bases then in parts of what Bedorf (56) calls “misjudging recognition”: The problem that the actual recognition of a person (even in the way of person-centred care) would always overwrite what a person could possibly be and want.

### 4.3 The social reality status of dignity

From a sociological perspective, a person’s autonomy and dignity – even in the Kantian sense of the *Menschenwürde* - are not inherent qualities. The sociality established and maintained in care relationships cannot simply be linked to the clinical picture of the person in need of care, but is essentially related to the specific dynamics of interaction. This therefore also applies to the form in which the dignity of those in need of care is asserted, as well as that of carers. This draws attention to the practical conditions for the recognition and institutionalisation of respect for the dignity of both people with dementia and carers and other groups of actors. The notorious vagueness of the concept of dignity is therefore not only a problem of philosophical ethics (57), but conversely, ethics as one actor among many contribute to a practically effective concretisation in everyday organisational life. In this context, it is a difficult question to answer to what extent the administration of psychotropic drugs harms or benefits the autonomy of people in need of care. On the one hand, this is due to the fact that it is administered partly because the subjectivity of those in need of care is considered to be damaged and the administration of psychotropic drugs is supposed to be able to help them to exhibit behaviour that is socially expected to be based on free will. On the other hand, it is assumed that the administration of psychotropic drugs can in turn damage the subjectivity of those in need of care in such a way that their autonomy can also be restricted. The corridor for the legitimate administration of psychotropic drugs is then correspondingly narrow and notoriously controversial.

Sociology cannot solve ethical problems. However, its empirical research may help at least to indicate such problems.

## Data Availability

The raw data supporting the conclusions of this article will be made available by the authors, without undue reservation.

## Footnotes

1 In the following, I use the word in the theoretically undemanding sense of a socially generalised interpretation of a situation.
